# The dielectric relaxation behavior induced by sodium migration in the Na_2_CoSiO_4_ structure within a three-dimensional Co–O–Si framework

**DOI:** 10.1039/d0ra04912c

**Published:** 2020-07-22

**Authors:** Kawthar Trabelsi, Karim Karoui, Abdelfattah Mahmoud, Jérôme Bodart, Frédéric Boschini, Abdallah Ben Rhaiem

**Affiliations:** Laboratory LaSCOM, University of Sfax BP1171 3000 Sfax Tunisia abdallahrhaiem@yahoo.fr; GREENMAT, CESAM, Institute of Chemistry B6, University of Liège 4000 Liège Belgium

## Abstract

The disodium cobalt(ii) orthosilicate material (NCS) has been synthesized using improved solid-state (NCS-SS) and co-precipitation (NCS-CP) methods of synthesis. The Rietveld refinement of the XRD pattern of Na_2_CoSiO_4_ has demonstrated an orthorhombic crystal system with the space groups *Pna*2_1_ and *Pbca* for NCS-SS and NCS-CP respectively. The elemental mapping of microstructures by scanning electron microscopy-energy dispersive spectroscopy (SEM-EDS) showed the porous morphology and the homogenous particles of the Na_2_CoSiO_4_ powders. Their dielectric properties were measured in the frequency and temperature ranges of 0.1–10^6^ Hz and 383–613 K respectively. Different dielectric relaxation phenomena associated with the Na^+^-ion migration through different paths were displayed in relation with the temperature and frequency. The decrease and increase in the dielectric properties were found to be dependent on the formation of short-range ordered structure formed after the migration of Na^+^-ions. In the present work, an attempt has been made to study the relation between the structural properties and the dielectric process. Thus, interesting insights into the transport behavior of Na^+^-ions in different chemical environments were obtained. This in turn provides an effective procedure to probe the relationship between the diffusion pathway of Na^+^-ions and the dielectric response.

## Abstract 2

A disodium cobalt(ii) orthosilicate material has been synthesized using improved solid-state and co-precipitation methods of synthesis. The Rietveld refinement of the XRD pattern of Na_2_CoSiO_4_ demonstrated an orthorhombic crystal system.

## Graphical abstract

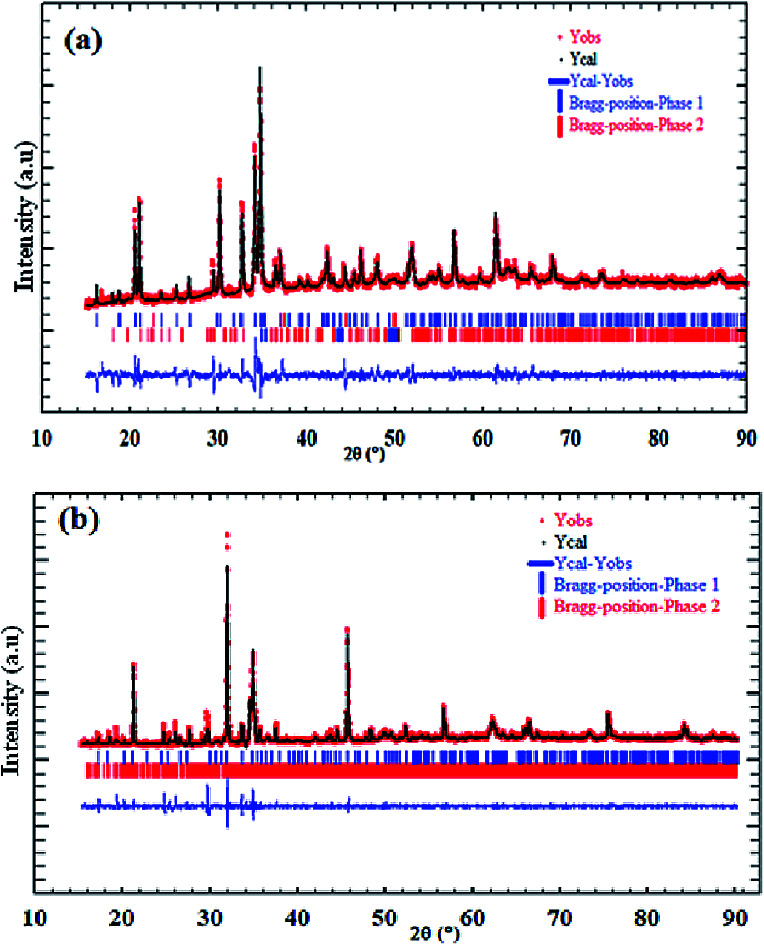

## 1. Introduction

Recently, considerable attention has been paid to Na-ion batteries (NIBs) with high energy density, low price and toxicity, relatively low self-discharge, good chemical stability, good mechanical stability and dielectric properties.^1–5^ Additionally, sodium is located below lithium in the periodic table, thus, such physical and chemical properties as ionic charge, electronegativity and electrochemical reactivity are very similar,^6–8^ which makes NIBs an attractive alternative to lithium-ion batteries (LIBs). More importantly, there are large numbers of resources of Na and Si in the Earth's crust. Na_2_CoSiO_4_ exhibits significantly lower polarization than Na_2_FeSiO_4_ and Na_2_MnSiO_4_. Much like Li_2_CoSiO_4,_ sodium cobalt-based orthosilicate (Na_2_CoSiO_4_) tends to be a good choice to serve as the cathode material in NIBs.^9^ WU Shun-qing *et al.*^10^ proved that changing the amount of Na in Li_2_CoSiO_4_ has a great influence on the structural properties. More precisely, this change is due to the presence of sodium and the distortion of its local environment, especially the SiO_4_ and CoO_4_ tetrahedra. Accordingly, we can notice that the sodium-ion migration and its environment are responsible for the physical proprieties which are significant for the cathode materials.^11,12^ The polymorphism of the cristobalite is porous while the arrangement of the transition metal silicate brings about large tunneling sites that can incorporate the diffusion of the cations.^13,14^ Disodium cobalt orthosilicate has attractive properties such as a high structural stability, a high Na^+^ diffusion of 8.0 × 10^−12^ cm^2^ s^−1^ at 300 K (ref. 15) and a good electrical conductivity which is reported to be purely ionic.^16^ The Na^+^ diffusion indicates a very low activation barrier and a 3D network of diffusion pathways through the SiO_4_ and MO_4_ frameworks,^17^ which both suggest favorable Na^+^ intercalation kinetics.

The dielectric permittivity, the dielectric loss and the electric modulus must be carried out in order to demonstrate the migration of sodium-ion in broad temperature and frequency ranges as well as to obtain a better understanding of the relaxation mechanism. Nevertheless, only few researchers have studied the dielectric properties of electrodes.

Finding a relationship between the composition, the structure and the dielectric properties should be of considerable interest. Therefore, the aim of this work is (a) to reexamine the structure of Na_2_CoSiO_4_ by the improved solid-state and co-precipitation methods, (b) to discuss its crystal chemical characteristics and its microstructure morphologies, and (c) to investigate the relaxation behavior of the Na^+^-ion migration as a function of the temperature, the frequency and the synthesis methods.

## 2. Experimental

### 2.1. Synthesis of Na_2_CoSiO_4_

In this work, the Na_2_CoSiO_4_ (NCS) material was prepared through the improved solid-state (NCS-SS) and co-precipitation (NCS-CP) methods.

The Na_2_CoSiO_4_ (NCS-SS) was synthesized for the first time by the improved solid-state method. The reaction scheme was the following:

1 6Na_2_CO_3_ + 6SiO_2_ + 2Co_3_O_4_ → 6Na_2_CoSiO_4_ + 6CO_2_ + O_2_

Na_2_CO_3_ and Co_3_O_4_ were used as starting materials for sodium and cobalt sources respectively. Na_2_CO_3_ (Sigma-Aldrich, 99%) and Co_3_O_4_ (Sigma-Aldrich > 97%) were first mixed in ethanol under magnetic stirring at 50 °C for 5 hours. Then, the silicon source (SiO_2_) (Sigma-Aldrich, 99.9%, 0.5–10 μm) was added to the suspension. The temperature increased to 80 °C to evaporate the ethanol and the water. Next, a few drops of nitric acid were slowly added to the above mixture. After that, a homogeneous suspension was formed and kept stirring until it yielded the mixed precursor. The resulting suspension was dried at 350 °C for 12 h to avoid ambient moisture and gases, especially CO_2_ evaporation. The grey powder was sintered at 900 °C for 9 hours under an argon atmosphere to avoid contact with humid air and to form the blue NCS-SS sample. On the other hand, Na_2_CoSiO_4_ was prepared according to the synthesis protocol shown by Joshua Treacher *et al.*^15^ using the co-precipitation synthesis route. NaOH (Sigma-Aldrich > 98%), CoCl_2_ (Sigma-Aldrich > 98%) were used as sodium and cobalt sources and TEOS (tetraethylorthosilicate) (Sigma-Aldrich, 98%) as a silicon source. The resulting dry residues were washed with ethanol and distilled water to remove the presence of Cl^−^. The blue precipitate was dried at 80 °C for 12 h. The prepared powder was sintered eventually at 600 °C for 8 h under an argon atmosphere to obtain the NCS-CP material.

### 2.2. Characterization techniques

The phase formation was identified by the X-ray powder diffraction technique using Bruker D8 Discover Twin-Twin with an advance diffractometer in Bragg–Brentano geometry with Cu Kα radiation (*λ* = 1.5406 Å, 10° ≤ 2*θ* ≤ 90°). Refinements were carried out using the FullProf program based on the Rietveld method.^18^ To investigate the morphology of the prepared samples, the scanning electron microscope (XL30 FEG ESEM, FEI) was used with an accelerating voltage of 15 kV under high vacuum. Dielectric impedance measurements were determined using the double platinum electrode configuration of Solartron SI-1260 in the frequency range of 0.1–10^6^ Hz and the 383–613 K temperature range.

## 3. Results and discussions

### 3.1. X-ray diffractions

**Fig. 1:**
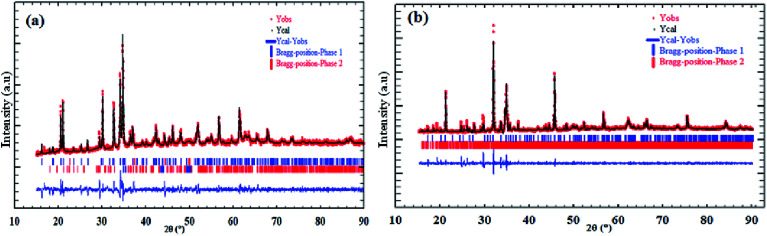
Graphical results of the Rietveld refinement of synthesized Na_2_CoSiO_4_ by (a) ameliorate solid state method denoted NCS-SS, (b) co-precipitation method denoted NCS-CP.

The refinement results of X-ray diffraction patterns of the synthesized Na_2_CoSiO_4_ (NCS-SS and NCS-CP) at room temperature are shown in Fig. 1(a) and (b) for NCS-SS and NCS-CP. The Rietveld refinement results are in agreement with the previously reported data.^15^ The small difference between the calculated/experimental patterns and the low values of the agreement parameters confirms the successful refinement of the experimental data. The main X-ray diffraction peaks were assigned to the Na_2_CoSiO_4_ material for the two samples. In addition, the peaks were sharp indicating the high degree of crystallinity of the synthesized NCS samples.

Overall, Na/Li orthosilicate materials were indexed in primitive systems.^19^ NCS-SS was indexed successfully on the basis of an orthorhombic unit cell with the *Pna*2_1_ space group. The corresponding cell parameters obtained from the data are as follows: *a* = 10.9549(7) Å, *b* = 5.2523(3) Å, *c* = 7.0440(6) Å, *α* = *β* = *γ* = 90°, *V* = 405.29(5) Å^3^ and *Z* = 4 with a reliability factor of *χ*^2^ = 1.8. The small peaks correspond to the impurity phase δ-Na_2_Si_2_O_5_ (3.6%).^20^ While NCS-CP has the same orthorhombic crystal structure, its space group is *Pbca* and its cell parameters correspond to: *a* = 10.3176 (2) Å, *b* = 14.6019 (7) Å, *c* = 5.1558 (2) Å, V = 771.85(3) Å^3^ and *Z* = 8 with a reliability factor of *χ*^2^ = 1.48. Based on the refinement, the relative amount of the NCS-CP phase is 96.88%. However, the relative amount of the γ-Na_2_Si_2_O_5_ impurity is 3.18%. Na_2_Si_2_O_5_ is frequently the minority impurity in the NCS compounds. The refined structural parameters of NCS-SS and NCS-CP and their impurities are summarized in Table 1. Both factors *R*_wp_ and *R*_B_ which are the fit factors are reasonably small, indicating that the phases of sodium orthosilicate are appropriate structural models for Na_2_CoSiO_4_. The final atomic coordinates and the isotropic displacement parameters are listed in Table 2, while the bond angles and distances are given in Tables 3 and 4.

**Table 1:** Crystal data of the Na_2_CoSiO_4_ compound and the detected impurities

Formula	NCS-SS	δ-Na_2_Si_2_O_5_	NCS-CP	γ-Na_2_Si_2_O_5_
Crystal system	Orthorhombic	Monoclinic	Orthorhombic	Monoclinic
Space group	*Pna*2_1_	*P*12_1_/*c*_1_	*Pbca*	*C*12/*c*_1_
Formula units (Å)	*a* = 10.9549(7)	*a* = 8.3993(2)	*a* = 10.3176(2)	*a* = 33.3766(11)
	*b* = 5.2523(3)	*b* = 12.083(3)	*b* = 14.6019(7)	*b* = 14.0877(5)
	*c* = 7.0440(6)	*c* = 4.843(1)	*c* = 5.1558(2)	*c* = 26.1507(7)
	*α* = *β* = *γ* = 90°	*α* = *γ* = 90°, *β* = 90.37(3)	*α* = *β* = *γ* = 90°	*α* = *γ* = 90°, *β* = 108.60°
*R*_p_ (%)	27.6	27.6	25.6	25.6
*R*_wp_ (%)	25.6	25.6	23.6	23.6
*R*_exp_ (%)	19.10	19.10	19.37	19.37
*R*_B_	3.462	2.245	2.384	3.129
*R*_f_	2.679	2.046	2.047	3.117
*χ* ^2^	1.79	1.79	1.48	1.48
%	96.4	3.6	96.88	3.18

**Table 2:** Fractional atomic coordinates and equivalent isotropic displacement parameters of Na_2_CoSiO_4_

Atom	Wyck	*x*	*y*	*z*	*O*_cc_
**NCS-SS**					
Co(1)	4*a*	0.15484	0.25746	0.63431	1
Si(2)	4*a*	0.40688	0.26700	0.10603	1
Na(1)	4*a*	0.17294	0.23567	0.10603	1
Na(2)	4*a*	0.40630	0.26710	0.36897	1
O(1)	4*a*	0.32064	0.24200	0.69309	
O(2)	4*a*	0.09690	0.44781	0.40866	
O(3)	4*a*	0.04951	0.28720	0.88692	
O(4)	4*a*	0.14226	0.88014	0.57608	

**NCS-CP**					
Co(1)	8*c*	0.97150	0.81450	0.70095	0.5
Si(1)	8*c*	0.97150	0.81450	0.70095	0.5
Co(2)	8*c*	0.26550	0.05584	0.27810	0.5
Si(2)	8*c*	0.26550	0.05584	0.27810	0.5
Na(1)	8*c*	0.48810	0.06810	0.68160	0.5
Na(2)	8*c*	0.50450	0.04080	0.77420	0.5
Na(3)	8*c*	0.74650	0.31280	0.18090	0.5
Na(4)	8*c*	0.79510	0.30730	0.37460	0.5
O(1)	8*c*	0.06560	0.91190	0.80300	
O(2)	8*c*	0.01920	0.78010	0.42380	
O(3)	8*c*	0.23960	0.95950	0.12760	
O(4)	8*c*	0.79130	0.83870	0.84560	

**Table 3:** Selected distances of Na_2_CoSiO_4_ (Å)

NCS-SS		NCS-CP	
Bond distances (Å)		Bond distances (Å)	
Co1–O1	1.865	Co1–O4	2.034
Co1–O2	1.982	Co1–O1	1.801
Co1–O3	2.127	Co1–O2	1.863
Co1–O4	2.028	Si1–O2	1.593
Si1–O1	1.603	Co2–O1	1.810
Si1–O2	1.693	Co2–O3	1.817
Si1–O3	1.590	Si2–O4	1.767
Si1–O4	1.621	Si2–O3	1.629

**Table 4:** Selected angles of Na_2_CoSiO_4_ (°)

NCS-SS		NCS-CP	
Atoms (1, 2, 3, 4)		Atoms (1, 2, 3, 4) with M = Co/Si	
O1–Co1–O4	93.93	O2–M1–O4	98.29
O3–Si1–O2	80.80	O4–M2–O1	96.78
O1–Si1–O2	90.78	O1–M2–O3	89.41
Co1–O1–Na2	99.34	O1–Na1–O3	69.44
Co1–O2–Na2	97.78	O3–Na1–O1	99.74
Co1–O4–Na2	95.16	O4–Na4–O2	96.29
Na1–O4–Na2	92.28	M2–O1–Na2	92.52
O1–Co1–O3	110.24	M2–O1–Na1	94.31
O2–Co1–O4	107.99	M2–O1–Na1	88.32
O2–Co1–O3	117.40	M1–O1–Na1	75.23
O4–Co1–O3	101.78	M1–O2–Na4	86.58
O1–Co1–O2	120.79	M2–O3–Na4	82.45
O3–Si1–O1	127.11	M2–O3–Na1	93.79
O3–Si1–O4	110.48	Na4–O3–Na1	71.28
O1–Si1–O4	122.20	Na3–O3–Na1	81.88
O4–Si1–O2	103.69	M1–O3–Na3	81.88
O3–Na1–O4	119.39	O2–M1–O1	110.16
O4–Na2–O2	116.49	O2–M1–O2	103.76
O4–Na2–O1	141.68	O2–M1–O4	131.77
O2–Na2–O1	101.44	O1–M1–O2	105.25
Si1–O1–Co1	138.50	O1–M1–O4	104.39
Si1–O1–Na2	120.95	O3–M2–O4	121.76
Si1–O2–Co1	128.05	O3–M2–O1	114.62
Si1–O2–Na2	115.50	O3–M2–O3	111.18
Si1–O3–Na1	130.89	O4–M2–O3	117.13
Si1–O3–Co1	118.66	O1–Na1–O1	104.77
Na1–O3–Co1	105.06	O4–Na3–O3	102.86
Si1–O4–Co1	123.80	O4–Na3–O4	119.65
Si1–O4–Na1	105.41	O3–Na3–O4	134.20
Si1–O4–Na2	129.24	O3–Na4–O4	147.93
Co1–O4–Na1	104.97	O3–Na4–O2	106.07
		M1–O1–M2	134.75
		M1–O1–Na2	120.60
		M1–O1–Na1	104.18
		M1–O1–Na1	125.19
		M2–O1–Na1	106.90
		M1–O2–M1	134.88
		M1–O2–Na4	137.91
		M2–O3–M2	125.72
		M2–O3–Na4	151.79
		M2–O3–Na3	125.03
		M2–O3–Na1	104.50
		M2–O3–Na3	107.73
		M2–O4–Na3	117.06
		M2–O3–M1	108.80
		M2–O3–Na4	134.27
		M2–O3–Na3	128.42
		Na3–O3–M1	120.68
		M1–O3–Na4	102.45

### 3.2. Description of the NCS structures

Na_2_MSiO_4_ (M = Fe, Mn, Co) is classified among the C-type compounds. It crystallized in the three-dimensional (3D) frameworks of the CoO_4_ and SiO_4_ tetrahedral units.^21^ The structure of Na_2_CoSiO_4_ deviated from the orthorhombic arrangement because of the different sizes of the Na, Co and Si atoms and their tetrahedral coordination. The powder pattern of Na_2_CoSiO_4_ indicates that it is isostructural with the Na_2_BeSiO_4_ structure.^22,23^

The structure can be considered as a ‘stuffed’ cristobalite with corner-sharing alternate transition metal and silicon tetrahedral units. In general, the orthosilicates are characterized by tetrahedral anion units, in particular (SiO_4_)^4−^, covalently bonded to the MO_4_ polyhedra.^24,25^ The relative orientation of the tetrahedra and the degree of corrugation in the layers differ considerably according to the modification.

**Fig. 2:**
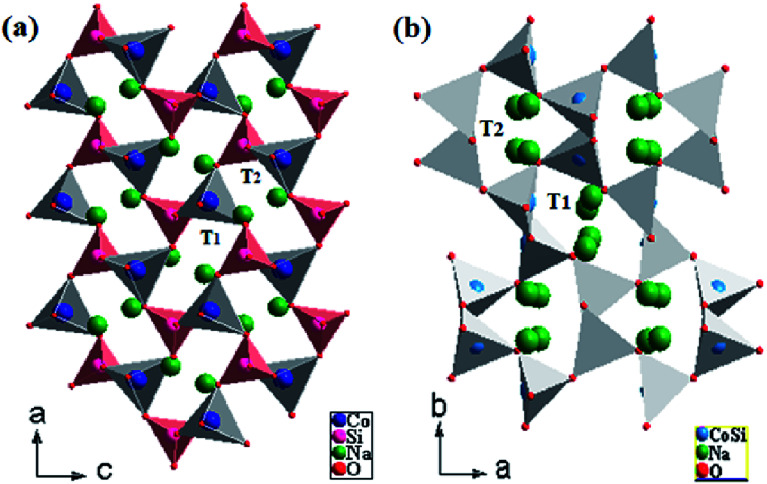
Na_2_CoSiO_4_ structures as a ‘stuffed cristobalite’ with corner sharing CoO_4_ tetrahedra connected *via* SiO_4_ tetrahedra (a) NCS-SS and (b) NCS-CP.

The *Pna*2_1_-Na_2_CoSiO_4_ structure can be viewed as a build-up of infinite zig–zag paths alternating (CoO_4_)^4−^ and (SiO_4_)^4−^ in the (*c*, *b*) plan. These tetrahedra built up a network with channels along the a axis in which the Na^+^-ions were arranged as represented in Fig. 2(a). In the periodic structure, half the CoO_4_ and SiO_4_ tetrahedra pointed along (100), while the other half pointed in the opposite direction (−100) in an alternate manner and were linked only by sharing corners. The mechanism process was induced by the parallel displacement of Na^+^-ions along the *c* axis. It consists of double chains of deformed (CoO_4_)^4−^ tetrahedra that were parallel-propagated in the (*a*, *c*) plan and interlinked partially by corner-sharing. Each (SiO_4_)^4−^ group was thus surrounded by four (CoO_4_)^4−^ tetrahedra and *vice versa*, ultimately forming cavities. The average values of the bond lengths of Si–O and Co–O in the (SiO_4_)^4−^ and (CoO_4_)^4−^ tetrahedra are 1.6465 Å and 2.0025 Å respectively (Table 3). Moreover, it is clear that the average bond lengths of Co–O in (CoO_4_)^4−^ tetrahedra were much larger than those of Si–O in the (SiO_4_)^4−^ tetrahedra. Na(1) and Na(2) ions occupied the tetrahedral sites located between two [SiO_4_–CoO_4_]^4−^ layers. Other paths with longer Na–Na hop distances should yield high migration for batteries around 3 Å. Regarding the Na(1) and Na(2), they were arranged along (100). Moreover, it is very important to note that Na(1) and Na(2) were trapped in two antiparallel cavities (T_1_ and T_2_) (Fig. 2(a)). This result would have a great influence on the migration process of Na^+^-ions.

The *Pbca*-Na_2_CoSiO_4_ is similar to the structure published by J. C. Treacher *et al.*^15^ The positions of Co^2+^, Si^4+^ cations in NCS-CP show that silicon and cobalt occupied the same site (Fig. 2(b)). Na^+^ cations were stuffed onto the vacant tetrahedral sites. All tetrahedra were not oriented in the same way. The results presented here demonstrate that future work should consider how to exploit the importance of cavities. Since the tetrahedra around the Na atoms only shared corners with neighboring Na cavity, and since the tetrahedra of the Si and Co atoms were fully occupied and smaller than the Na tetrahedra, the diffusion of Na^+^-ions must proceed through the unoccupied tetrahedral sites in the structure. Eventually, forming the parallel and antiparallel cavities (T_1_, T_2_) played the main role in the structure and clearly drove the system. In the present work, the different space groups could be expressed either Co and Si sources or the different sintered temperature (900° for NCS-SS and 600° for NCS-CP).

### 3.3. Scanning electron microscopy

**Fig. 3:**
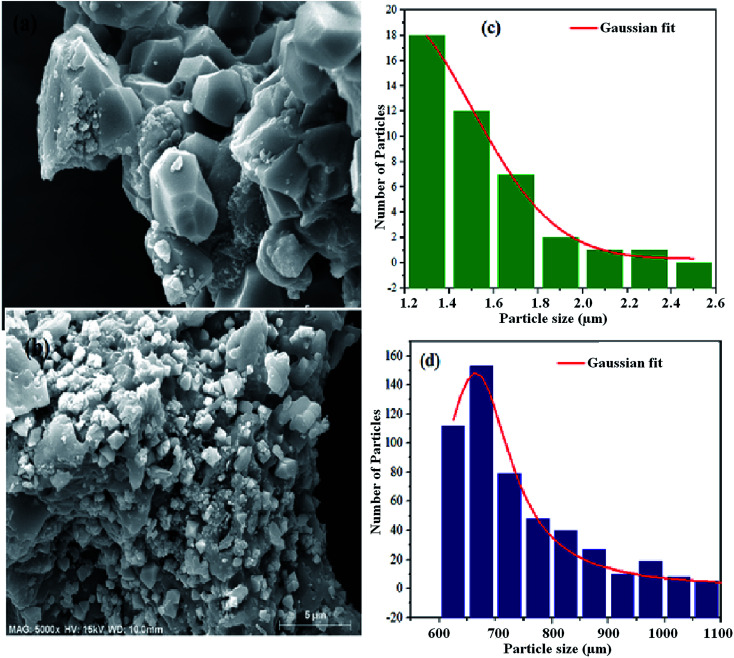
SEM micrograph of the fractured surface of NCS-SS (a) and NCS-CP (b) samples heat treated at 900 °C and 600 °C under argon. The corresponding particle size histograms (c and d) are presented in the right of the figure.

Besides the crystallographic structure, the morphological features are important to reinforce and bring out the dielectric mechanism of the material. Thus, we have studied the morphological characteristics of the prepared powders. The SEM micrographs of the NCS-SS and NCS-CP are presented in Fig. 3. The SEM images of the two samples were clearly constituted of an agglomeration of the small particles which led to sub-micrometer particles. This could be due to the relatively high calcination temperature (900 °C) and (600 °C) for NCS-SS and NCS-CP respectively. The measured sizes of the particles are 1.4 μm for NCS-SS and 0.7 μm for NCS-CP. Furthermore, in both Na_2_CoSiO_4_ samples, we could observe the presence of small-sized particles defined as secondary particles. Consequently, the presence of these particles may improve the charge storage through the reduction of migration path lengths.^26^ Hence, the formation of micro-sized particles, hollow structure and morphology density are beneficial as electrode material for secondary batteries.^27^

**Fig. 4:**
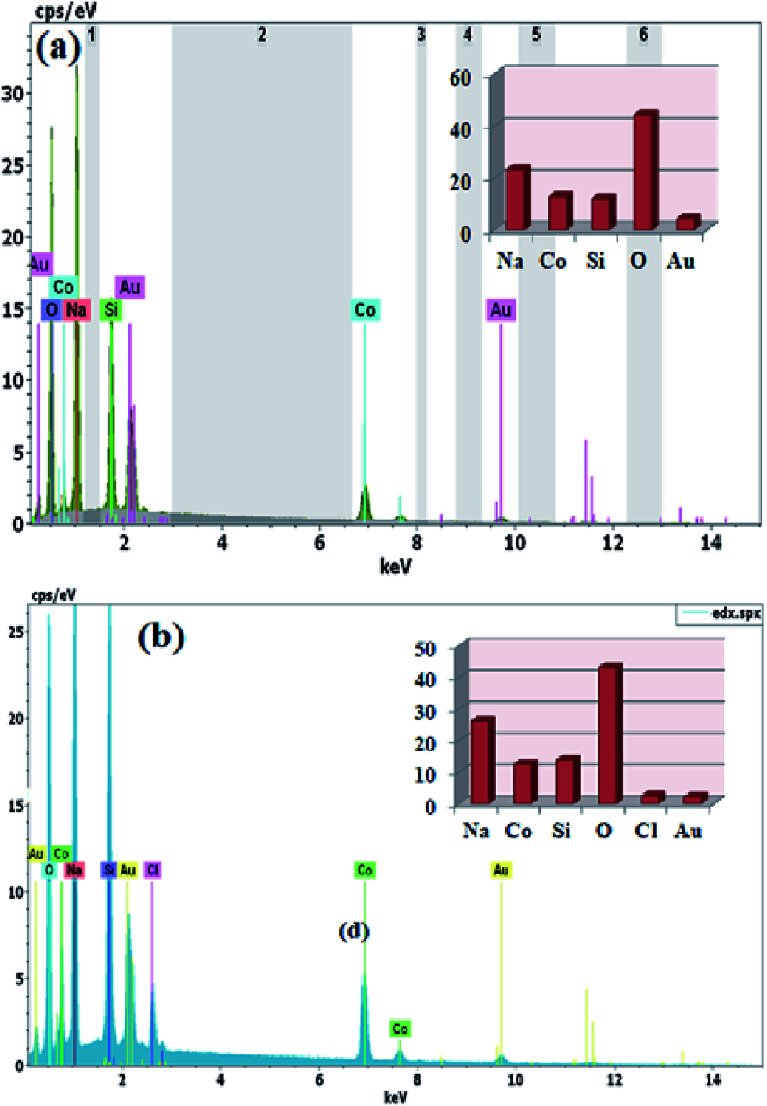
EDX spectra of the fractured surface of NCS-SS (a) and NCS-CP (b) samples sintered at 900 °C and 600 °C, respectively.

On the other hand, the SEM and EDS link the surface morphology to its chemical composition. The EDS spectra were taken at several points on the surface of the samples. In Fig. 4(a) and (b), the existing elements are illustrated in the EDS spectra. In order to measure the stoichiometry of each element, the EDS analysis was carried out. The individual atoms such as Na, Co and Si were observed in the EDS spectra suggesting homogeneous distribution of the elements. Moreover, the ratios of atoms for Na, Co, Si and O were very close to the stoichiometric ratio of both Na_2_CoSiO_4_ samples which in turn supported the determination of the purity of the Na_2_CoSiO_4_ particles together with the powder XRD. It is interesting to note that Cl^−^-ion was directly observed with a small percentage in the surface layer of NCS-CP. The result is very close to its theoretical composition ratio 2 : 1 : 1.

### 3.4. Dielectric relaxation

**Fig. 5:**
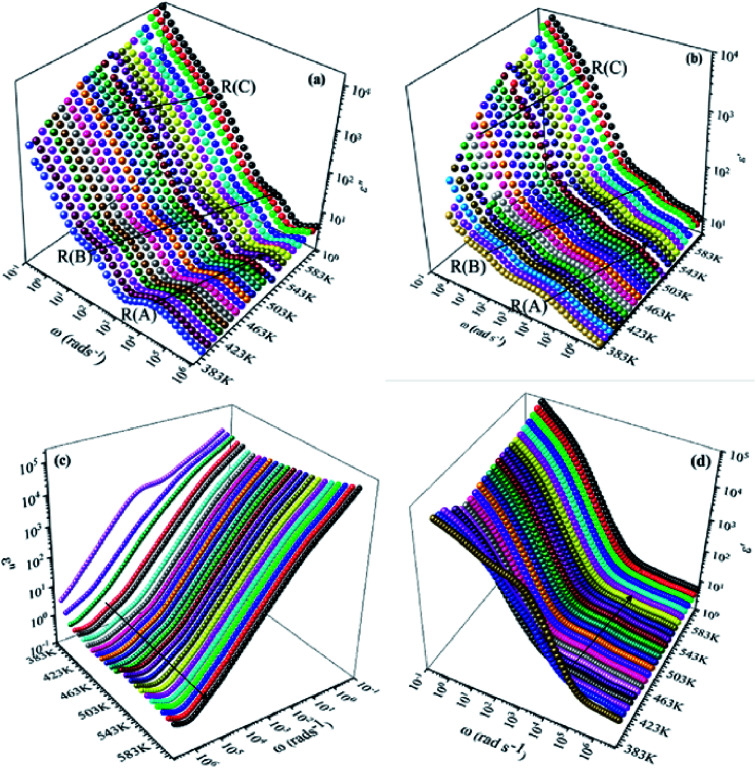
Dielectric loss *ε*′′ and dielectric constant *ε*′ as a function of frequency for NCS-SS (a and b) and NCS-CP (c and d) at various temperatures ranging from 383 K to 613 K with the interval of 10 K.

**Fig. 6:**
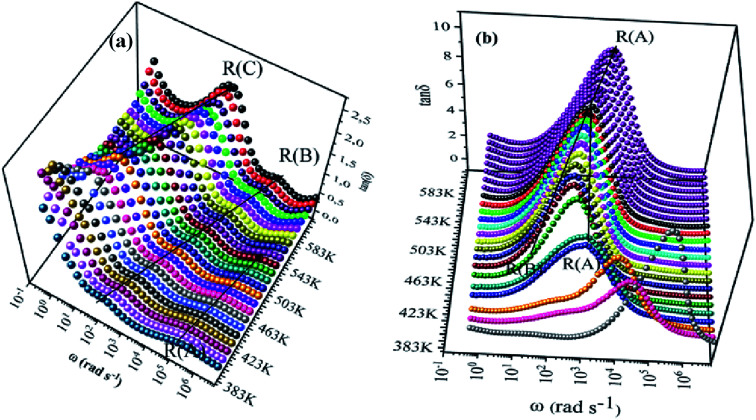
Frequency dependence of tan *δ* at various temperatures for NCS-SS (a) and NCS-CP (b) as a function of temperature.

We have adopted the permittivity formalism to study the relaxation mechanism and to explore the ion dynamics in the NCS compounds at different temperatures and frequencies. The real (*ε*′) and imaginary (*ε*′′) parts of the complex permittivity for NCS-SS and NCS-CP are plotted in Fig. 5(a)–(d) as a function of angular frequency *ω* (*ω* = 2π*f*) at various temperatures. Both *ε*′ and *ε*′′ attained a minimum value at high frequencies irrespective of the temperature. The mounting value of *ε*′ with the climb of temperature might be attributed to the rise in the process of space charge polarization. With the increasing frequency (when *ω* < 1/*τ*), dipoles began to lag behind the field and *ε*′ slightly decreased. When the frequency reached the characteristic frequency (*ω* = 1/*τ*), the dielectric constant dropped and demonstrated the relaxation process. At very high frequencies (*ω* ≫ 1/*τ*), dipoles could no longer follow the field. Thus, the increasing value of *ε*′ in the high and mid frequency regions was associated with the dipolar polarization (Na^+^-ions). The high values of *ε*′ at a relatively low frequency might be due to the extrinsic contribution (the space charge localization at NCS). It seems clear that NCS-SS presents three contributions. The two contributions at high and mid frequencies were shown by two types of charge carriers (Na^+^-ions) which were responsible to move only over short distances. This process was in agreement with the existence of two types of cavities in both NCS materials. The third contribution corresponded to the process of space charge polarization at low frequencies. At high frequency, NCS-CP has demonstrated a weak appearance of the relaxation peaks. According to Fig. 5(b) and (d), *ε*′′ increased with mounting temperature, which reveals the dipolar molecular dynamics at high and medium frequency ranges. Typically, the dielectric constant shifted toward high frequencies with the increase in temperature, exhibiting a thermally activated behavior. Moreover, the rise in temperature brought thermal agitation in the compounds, which can produce a deformation cavity.^28^ Consequently, the mobility of the ions increased. The corresponding variation of the dielectric loss as a function of the angular frequency has been investigated. Fig. 6(a) and (b) display the tan *δ* spectra. We can see that three clear peaks were detected in the frequency dependence of the dielectric loss for NCS-SS and two peaks one of which was too weak to be observed for NCS-CP. For the NCS-SS material, when the temperature climbed, the first relaxation (A) disappeared at high frequencies between 1 × 10^6^ to 3.8 × 10^4^ Hz. At mid frequency, the second relaxation (B) between 2.4 × 10^4^ and 1.4 × 10^3^ Hz spread to high frequencies when the temperature went up. Meanwhile, the third relaxation (C) appeared at a certain low frequency below 200 Hz and in a high temperature region. Since the relaxation maximum shifted monotonically to low temperatures with decreasing frequency, we could attribute this dielectric dispersion phenomenon to a thermally-activated process. The relaxation time for the medium and high frequency relaxations was very close. For NCS-CP, two clear peaks are observed in the frequency dependence of the dielectric loss as shown in Fig. 6(b). The first one occurred at mid frequency range 500–3700 Hz (relaxation B) and the second was presented at high frequency range 8 × 10^3^–5 × 10^5^ Hz (relaxation A). The relaxation peaks moved toward high frequencies with the increase in the temperature, indicating that the relaxation processes in both NCS samples are temperature dependent. In the main processes, the Na and O positions were quite dynamic, while the Co–Si network remained relatively undisturbed to maintain the stability of the structure as a function of temperature. Accordingly, understanding the distinction of the relaxation process between NCS-SS and NCS-CP provides a more comprehensive image of the system dynamics and the transport mechanisms. First, NCS-SS (with the *Pna*2_1_ space group) had lower local Na coordination symmetry than NCS-CP (with the *Pbca* space group) which was caused by the different silicon and cobalt sources.^29,30^ Second, the NCS-SS material presented two different charge carriers Na(1) and Na(2) in two equivalent cavities. However, NCS-CP presented four charge carriers Na(1), Na(2), Na(3) and Na(4) in two non-equivalent cavities.^31^ The Na^+^ environment in NCS-CP was more congested than NCS-SS, because of the duplication of Si and Co and the presence of Cl^−^.

We notice that the Coulombic attraction and the network are based on the increase in Na^+^, but the effect of the cation–cation repulsion and the steric effects can facilitate the jump of the Na^+^-ions along the canals. In this context, the above-listed results also imply that the Na-ions are less tight in NCS-CP than in NCS-SS, especially that the volume of NCS-SS (405.30 Å^3^) is lower than NCS-CP (771.74 Å^3^). Therefore, the connection of Na polyhedra leads to a flat energy landscape for sodium cations, which is beneficial for ion transport. As these sites are supposed to be metastable for the migration of sodium-ions, the migration energy barriers of sodium-ions depend on the crystallographic sites. In fact, the higher Na–O average bond distances (2.2587 Å) in NCS-SS with respect to in the NCS-CP ones (2.2632 Å) induces a decreasing of the attraction force between Na^+^ and O^2−^ and the ions becomes less attached to the crystal.

### 3.5. Activation energy

**Fig. 7:**
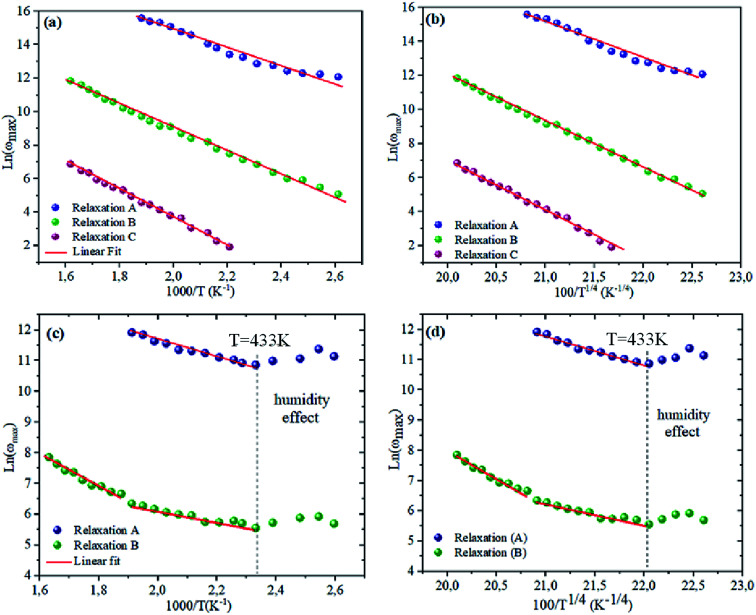
(a and c) dependence of relaxation frequency *ω* on temperature with ln(*ω*_max_) *vs.* 1000/*T*. The solid lines are the best-fitting results according to eqn (2). (b and d) The dependence of the relaxation frequency *ω* on temperature with ln(*ω*_max_) *vs.* 100/*T*^1/4^. The solid lines are the best-fitting results according to eqn (3).

To bring out the relaxation response in the prepared materials, it is necessary to find the activation energy of the relaxation process for each composition. Fig. 7(a) and (b) are plotted the variation of ln(*ω*_max_) as a function of the reciprocal of the temperature. A thermally-activated relaxation process is observed in both samples and confirms the semiconducting characteristic of samples. Notably, a humidity effect appears at around *T* = 533 K for NCS-CP sample.

The *ω*_max_ data were fitted using the Arrhenius law equation:^32^2
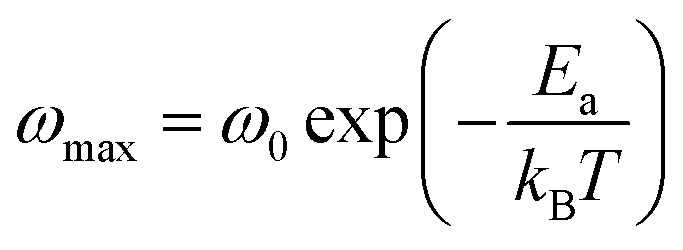
where *E*_a_ is the activation energy required for the thermally-activated process, *ω*_0_ represents the pre-exponential factor and *k*_B_ is the Boltzmann constant. The activation energy is presented in Table 5. It is notable that the activation energies of NCS-SS were higher than those of NCS-CP. The experimental data exhibits a deviation from the Arrhenius law in several materials, which shows the polaron relaxation related to localized charge carriers.^33–37^

**Table 5:** Activation energy obtained from the curves in Fig. 7(a) and (c)

Relaxation	NCS-SS	NCS-CP
Relaxation A	0.45 eV	0.25 eV
Relaxation B	0.59 eV	0.41 eV (at high temperature)
		0.18 eV (at low temperature)
Relaxation C	0.71 eV	

Moreover, a Mott's variable-range-hopping mechanism for polarons could fit better as described by eqn (3).^38^3
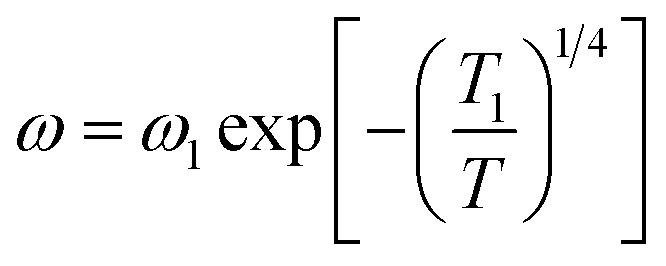
where *ω*_1_ and *T*_1_ are two fitting constants. The solid lines of this model are shown in the inset of Fig. 7(c) and (d). The values *T*_1_ for NCS-SS were 2.18 × 10^7^ K, 5.12 × 10^7^ K and 8.31 × 10^7^ K for relaxations (A), (B) and (C) respectively. The values for NCS-CP were determined to be 1.8 × 10^7^ K for relaxation (A) and 7.59 × 10^7^ K between 533 K and 613 K as well as 8.4 × 10^7^ K below 533 K for relaxation (B). The *T*^−1/4^ law confirmed the polaron relaxation in NCS samples.^11,39^ The quasiparticle (polaron) could move between different vacant sites.

The transition energy of a polaron can be expressed by the following equation:^11^

4 *W* = 0.25*k*_B_(*T*_1_)^¼^*T*^¾^

The hopping energy *W* calculated by eqn (4) increased from 0.15 eV at 383 K to 0.19 eV at 533 K for the relaxation (A), from 0.15 eV at 383 K to 0.22 eV at 613 K for the relaxation (B) and from 0.2 eV at 453 K to 0.25 eV at 613 K for NCS-SS. The sample NCS-CP exhibited the same behavior. The W values were found to be 0.21–0.26 eV below 513 K and 0.21–0.24 eV at high temperature for the relaxation (A) and 0.79–0.89 eV for the relaxation (B). The activation energy for the relaxation indicates that the relaxation mechanism for the two NCS materials is associated with polaron hopping based on charge carriers in order to account for the origin of a dielectric relaxation observed in NCS-SS and NCS-CP. These results hint that the sodium-ions over these sites probably pass from Na(4*a*) for NCS-SS and Na(8*c*) for NCS-CP to other sites.

### 3.6. Frequency and temperature dependence of the electric modulus formalisms

To emphasize the relaxation phenomena, a study of the complex modulus at various temperatures was carried out in the same frequency range. The electric modulus was employed to investigate the polaron relaxation and to avoid the dynamical aspects of the electrical transport phenomena in the NCS samples.^40^ The complex electric modulus (M*) could be calculated by the conversion formula (eqn (5)) using the complex dielectric constant (ε*), which is defined by:5
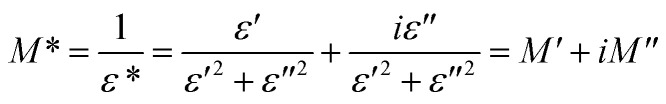
where 
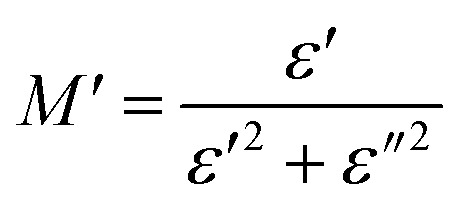
 is the real part of complex modulus and 
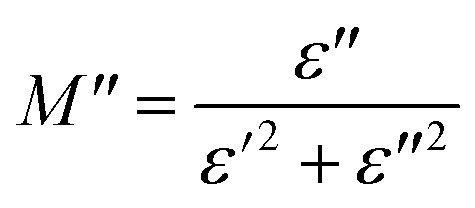
 is the imaginary part of the complex modulus.

**Fig. 8:**
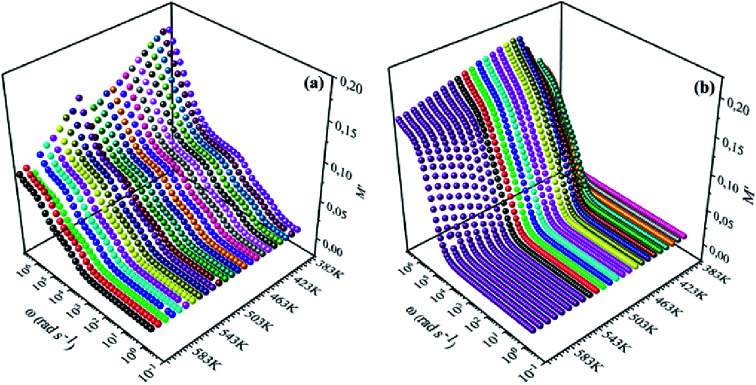
Frequency dependence of real of electric modulus (*M*′) for (a) NCS-SS and (b) NCS-CP at various temperatures.

Fig. 8(a), (b), 9(a) and (b) show the variation of the real and imaginary parts of the modulus as a function of angular frequency at various temperatures for NCS-SS and NCS-CP. At low frequencies, *M*′ exhibited very small values and tended to zero and, thus, suggesting the suppression of the electrode polarization effect.^41^ As the frequency increased, *M*′ climbed to a maximum asymptotic value defined as 
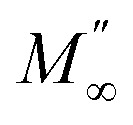
 due to the distribution of relaxation processes over a range of frequencies. Both samples produced a two-step decrease in *M*′ in the measured temperature range, while the NCS-CP peaks shifted to one step above 533 K. Correspondingly, *M*′′ showed two peaks in the same temperature range. The modulus spectra presented different relaxation mechanisms which depend upon temperature and frequency regions. We observe that NCS-SS appearing in *M*′′ along the temperature ranges. NCS-CP revealed two peaks, which shifted to a single peak at high temperature. To distinguish localized dielectric relaxation processes, R. Gerhardt proposed two ways: as far as NCS-CP is concerned, this material presented a pure conduction process (relaxation B), which could be seen as a relaxation peak observed in the frequency spectra of the imaginary component *M*′′ and no clear peak appeared in the corresponding plot of *ε*′ and *ε*′′, while for relaxation A, this sample (NCS-CP) showed weak peaks of *ε*′ and *ε*′′. This mixed process was related to the presence of two charges carriers (Na^+^). As regards NCS-SS, this material indicated a dielectric relaxation process as the relaxation peaks appeared in all the representations of *M*′′, tan *δ*, *ε*′ and *ε*′′.^42,43^ The peaks at low and high temperatures might be attributed to the existence of dissociate Na-ions or steady species caused by linked Na in NCS samples. Whilst, the peak overlap (above 533 K) is attributed either to cavity deformation or the two charge carriers Na^+^ are the same response at high temperatures in NCS-CP.^44,45^ The spectra of *M*′′ peaks at both high and mid frequencies suggest that two processes contribute to the diffusion of two Na charge carriers. One of these processes relaxed at the high frequency region but the contribution of the other process appeared as a peak in the mid frequency region. The values of 
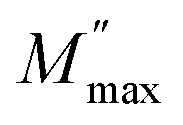
 shifted to the high frequency values with the increase in the temperature. The imaginary part of the electric modulus in different temperatures has been fitted with the function proposed by Bergman:^46^6
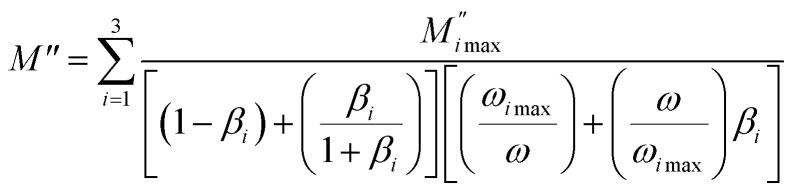
where the 
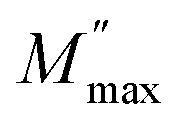
 is the maximum peak value of *M*′′, *ω*_max_ is the corresponding frequency and the index *β* indicates the degree of deviation from the Debye-type relaxation.

**Fig. 9:**
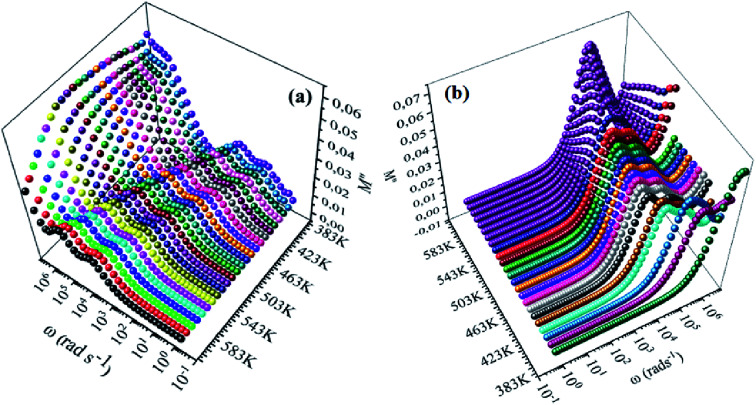
Frequency dependence of imaginary of electric modulus (*M*′′) for NCS-SS (a) and NCS-CP (b) at various temperatures.

**Fig. 10:**
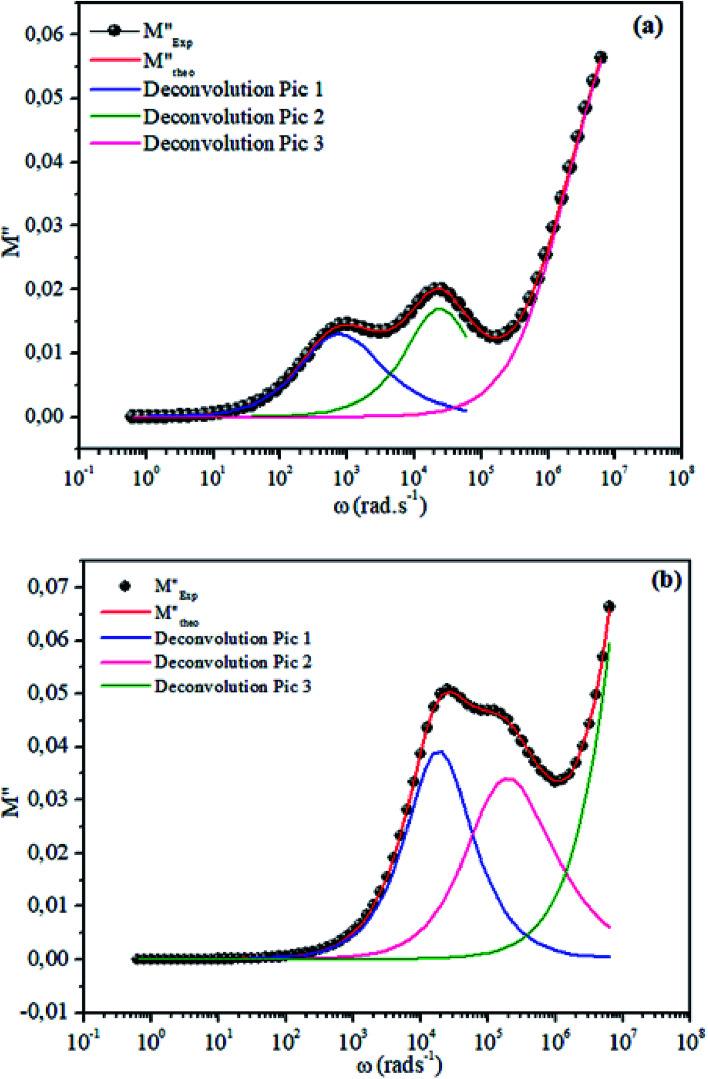
Deconvolution of experimental imaginary parts of impedance (*M*′′) as a function of angular frequency for NCS-SS (a) and NCS-CP (b) at 513 K used eqn (6).

**Fig. 11:**
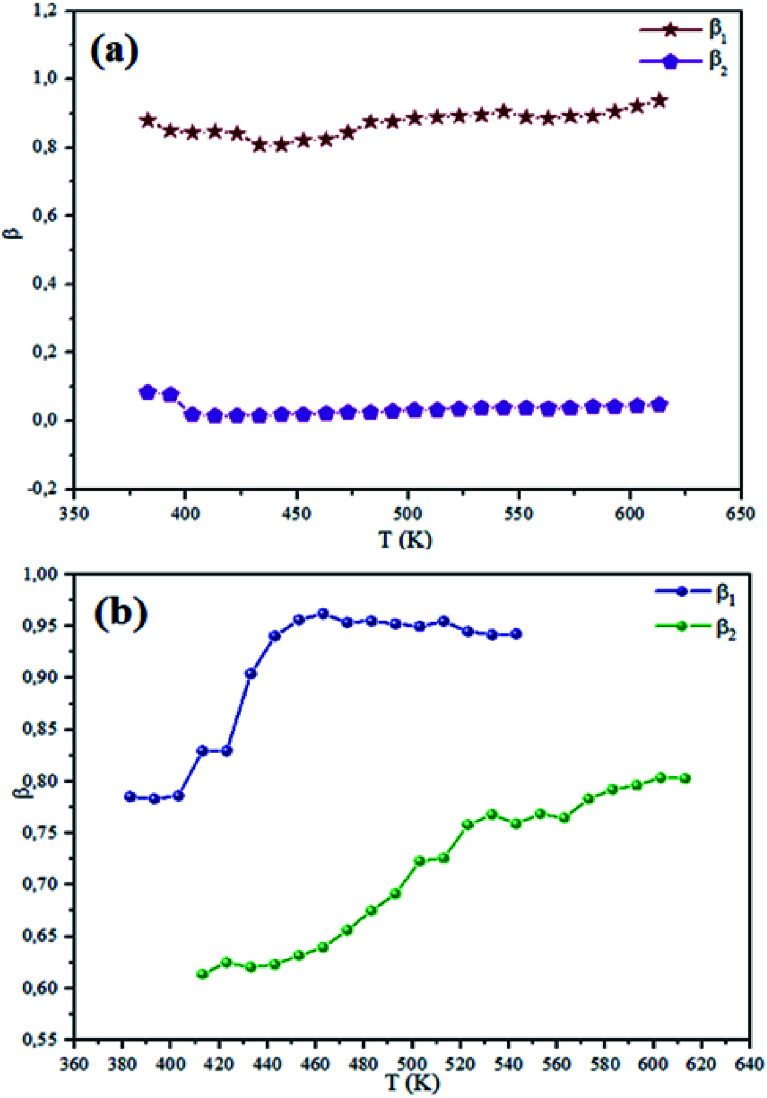
Temperature dependence of the NCS-SS (a) and NCS-CP (b) at various temperatures.

**Fig. 12:**
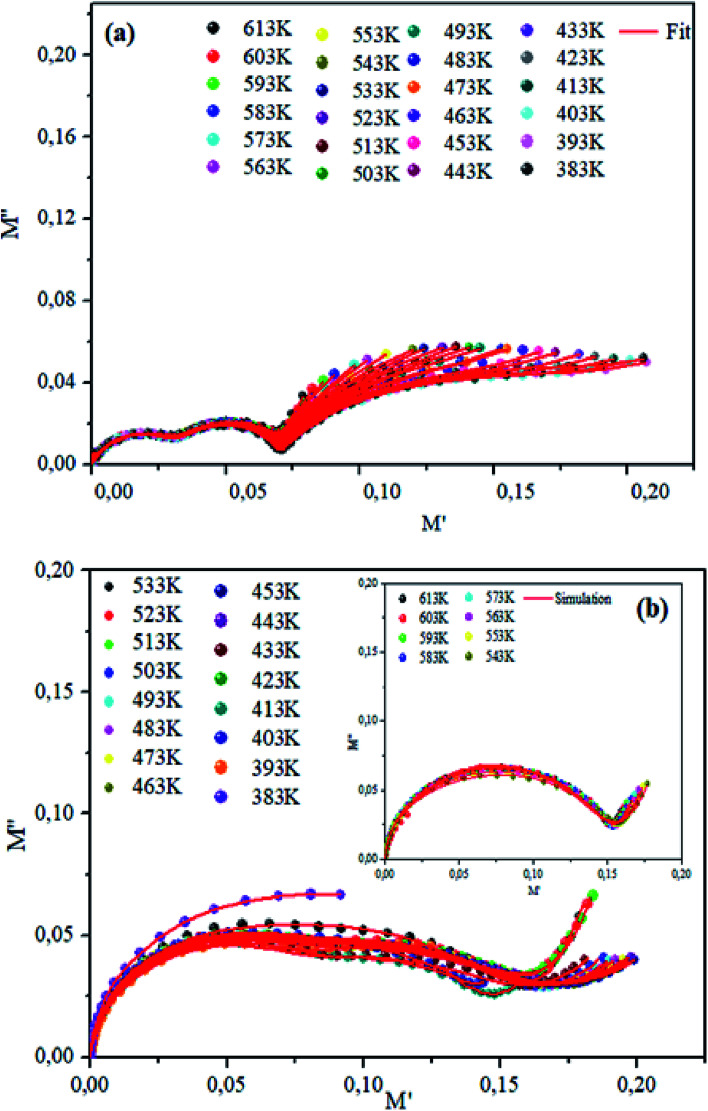
Plots of imaginary modulus *M*′′ *vs. M*′ real modulus for NCS-SS (a) and NCS-CP (b) at various temperatures.

**Fig. 13:**
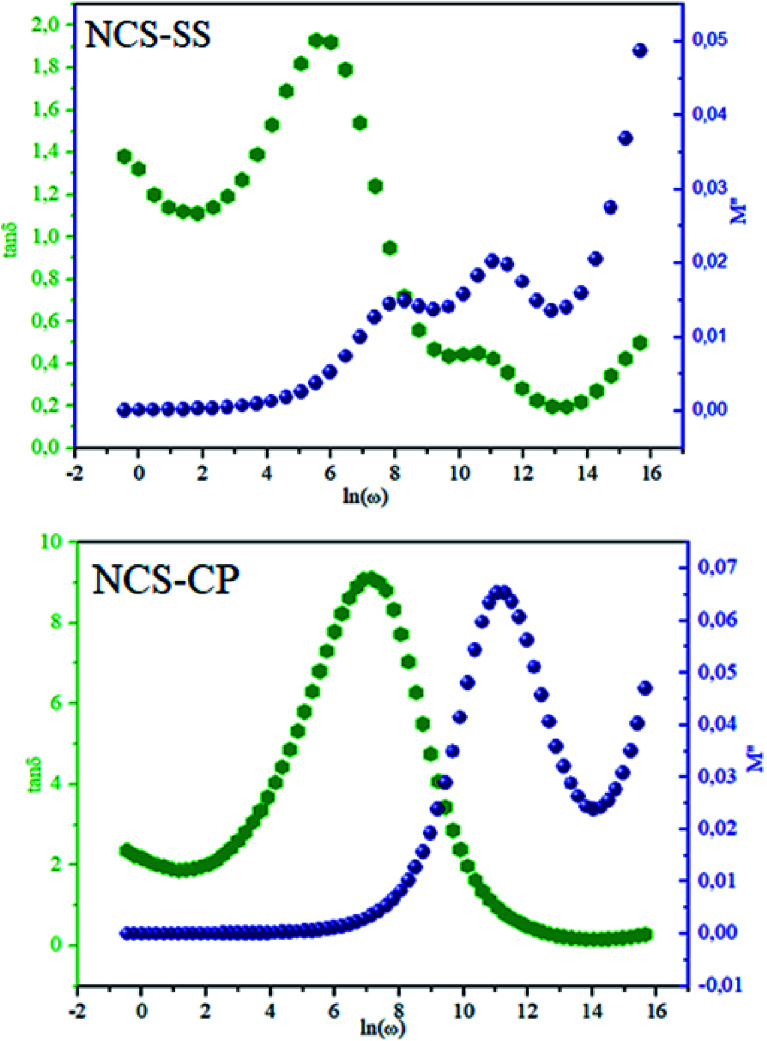
Frequency dependence of relaxation peaks, *M*′′ and tan *δ* for the two NSC compounds at 573 K.

**Fig. 14:**
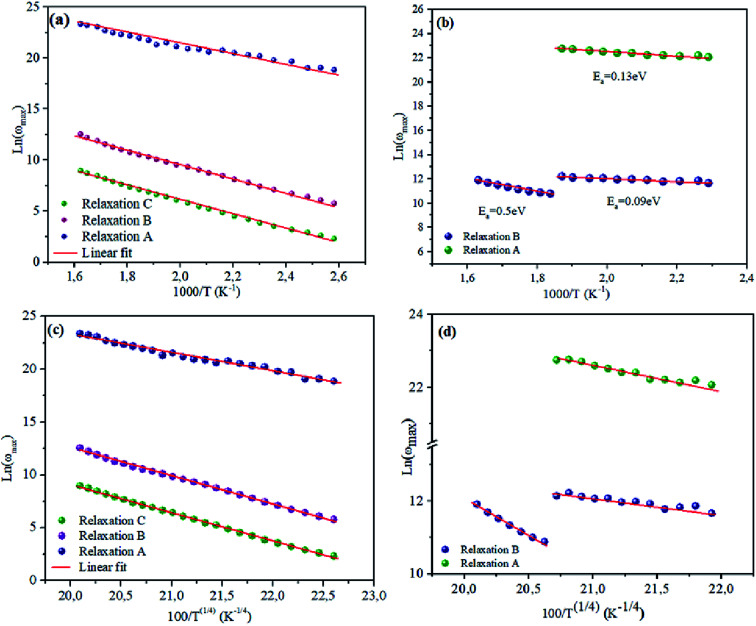
Dependence of relaxation frequency *ω* on temperature with ln *ω*_max_*vs.* 1000/*T* (a and b) and ln *ω*_max_*vs.* 100/*T*(1/4) (c and d) for NCS-SS and NCS-CP, respectively.

The theoretical fit and peak deconvolution (eqn (6)) of the experimental data are shown by solid lines in Fig. 10(a) and (b). It can be seen that the model fits the experimental data very well. The fit values of *β* (*β* < 1) suggest a non-Debye-type dielectric relaxation.^47^Fig. 11 shows the dependence of *β* values on the variety of temperatures. For NCS-SS, *β* varied slightly in the temperature range which indicates stability of the number of Na-ions available for short distance conduction. This result suggests that the polarons are stable in the dielectric process with the increase in the temperature. NCS-CP demonstrated higher values of *β*_2_ when the value of *β*_1_ increased to 0.96 below 453 K, and then slightly decreased to 0.92 as soon as the temperature went up to 613 K. The increase in *β* implies that there is a decrease in the number of sodium-ions available for short distance conduction where Na^+^-ions have a higher probability of being attracted by Cl^−^-ions.^48,49^ The complex electrical modulus of the NCS-SS sample (Fig. 12(a)) displays three semicircles. At low and mid frequencies, the arcs were perfectly overlapped into a single master curve, indicating that the underlying conduction mechanism remains the same, while at high frequency, we can seem a dispersion phenomena. As a result, the different contributions are linked to the transport mechanism of Na^+^ in or between cavities T_1_ and T_2_. On the other hand, NCS-CP displays only one semicircle between 543 K and 613 K, while it shows two discarded semicircles below 533 K (Fig. 12(b)). This result confirms that cavity is deformed at high temperature. The increase of temperature facilitates the long-range hopping movement of charge carriers Na^+^. This result is confirmed by the representations of *M*′′ and tan *δ* as a function of the logarithmic frequency measured at 573 K. We can see in Fig. 13 that the peak positions of *M*′′ and tan *δ* were more similar for NCS-SS than for NCS-CP. Therefore, we distinguish that, the peak positions between *M*′′ and tan *δ* were overlapped for NCS-SS. This indicates that long range and localized relaxations occur simultaneously. However, for NCS-CP, the two peak positions were dissimilar indicating a delocalized or a long-range transport mechanism.^50^ The dielectric relaxations processes suggest that the diffusion pathway of two sodium-ions could migrate over the same trajectories.^11^ The shift of the relaxation peak corresponds to the relaxation process towards higher frequencies with an increase in temperature which brings out a thermally-activated dielectric relaxation.^51^ At any temperature, the frequency *ω*_max_ defines the relaxation time *τω* = 1. In order to obtain the required activation energy for the dielectric relaxation process, the corresponding graphs of *M*′′as a function of *ω* and *T* for various values of *ω*_max_ are shown in Fig. 14. The temperature dependence of the characteristic relaxation frequency satisfies the Arrhenius law given by;^32^7
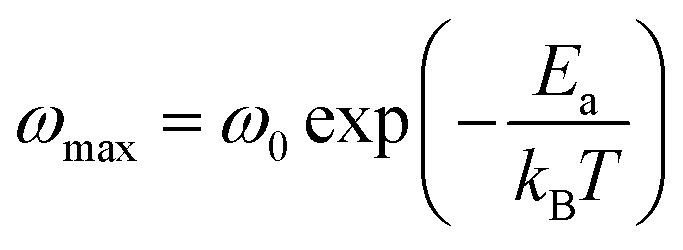


Consequently, it should be noted that *E*_a_ values which were obtained for the analysis of the results plotted in Fig. 14 were higher for NCS-SS than for NCS-CP. For the relaxation R(A), the calculated activation energies were 0.6 eV and 0.13 eV for NCS-SS and NCS-CP respectively. The *E*_a_ values for the relaxation R(B) were found to be 0.6, 0.59 eV and 0.5–0.09 eV for NCS-SS and NCS-CP respectively. The relaxation R(C) yielded 0.39 eV for NCS-SS. The variations of (*ω*_max_) obey the same Arrhenius law of eqn (3) (Fig. 14(c) and (d)). The parameter *T*_1_ was calculated to be 4.7 × 10^7^ K, 5.15 × 10^7^ K and 8.98 × 10^7^ K for R(A), R(B), R(C) respectively in NCS-SS. These values were equal to the ones obtained by the dielectric loss. The similar behavior of the relaxation process between the modulus and tan *δ* indicates that they originated from the same mechanism. However, NCS-CP pointed to very different values from those obtained by tan *δ*. The parameter *T*_1_ was calculated to be 0.16 × 10^7^ K for R(A) and 1.51 × 10^7^ K and 0.29 × 10^7^ K for R(B).

Generally, it is suggested that the dielectric relaxation might be due to such intrinsic contributions as the two types of charge carriers (sodium ions). In (Section 3.3), we pointed to the existence of the Cl^−^-ion in NCS-CP. In fact, Cl^−^ was located at the neighboring sites of Na and it had a different valence stability compared with O^2−^. Therefore, the only conclusion which could be drawn is that the Na site may be fully-occupied.^52^ Besides, the cationic distribution results from the energy minimization between: (1st) the electrostatic repulsions that tend to separate Na^+^-ions in the (*a*, *c*) plan, (2nd) the Na^+^–Co^2+^ repulsion through the common face between the NaO_4_ and CoO_4_ polyhedra, and (3rd) the electron–electron interaction in the cobalt layer. As all these parameters were very sensitive to the sodium content, various cationic distributions were observed all along the compositions.^53^ This brought the Na^+^-ions closer to the Co^2+^-ions and consequently generated a repulsive force between the two cations. Yet, the presence of Cl^−^ surface at the level of the compound NCS-CP could induce other electrostatic attractions which lead to lower activation energy and higher diffusion mechanism on the neighboring sites close to the Na migration route.^54^ We suppose, in consequence, that the dielectric relaxation is associated with the cavity.

## 4. Conclusions

Na_2_CoSiO_4_ samples have been successfully synthesized by the improved solid-state and co-precipitation methods. The NCS samples were found to crystallize in the orthorhombic symmetry with the *Pna*2_1_ and *Pbca* space groups for NCS-SS and NCS-CP respectively. The SEM images showed the appearance of particles of different sizes and forms due to the chemical process and sintered temperatures. The frequency-dependent plots of *M*′′, *ε*′, *ε*′′ and tan *δ* at different temperatures showed that there were two different relaxation regions in NCS-CP and three relaxations in NCS-SS. The low-frequency region was associated with the space charge, while the high-frequency region was associated with the confined charge carriers that moved over two different cavities. Furthermore, the dielectric proprieties of Na_2_CoSiO_4_ which were associated with the migration of the sodium-ions have been studied at various temperatures and frequencies. Consequently, we found a dielectric relaxation process for NCS-SS and a mixture of dielectric relaxation and conduction processes for NCS-CP. Interestingly, the transition energy was associated with a transition of the polaron between localized states with phonon assistance for NCS-SS and a transition of the polaron between delocalized states with phonon assistance for NCS-CP. The polaron relaxation process with the *T*^−1/4^ behavior at different temperatures is attributed to the charge carriers that can move between mixed valences of ions. The activation energy of NCS-SS was higher than that of NCS-CP. The different values of activation energies deduced from different formalisms suggest that the same charge species were responsible for the relaxation of NCS-SS; however, for NCS-CP, the different values of activation energies were caused by the presence of surfaced Cl^−^ and cavity (T_2_) depending on temperature. We also found that for this sample, a diffusion process at high temperature was associated to the displacement of Na-ions to the cavity equivalent to T_1_. This gives an insight into the diffusion behavior of Na-ions at different chemical environments and proves the previously-mentioned results to build the relationship between the migration of sodium-ions and the dielectric relaxation for sodium-based electrode materials.

## Conflicts of interest

There are no conflicts to declare.

## Supplementary Material
